# Immunomodulation by lipid emulsions in pulmonary inflammation: a randomized controlled trial

**DOI:** 10.1186/s13054-015-0933-6

**Published:** 2015-05-12

**Authors:** Matthias Hecker, Tomke Linder, Juliane Ott, Hans-Dieter Walmrath, Jürgen Lohmeyer, István Vadász, Leigh M Marsh, Susanne Herold, Martin Reichert, Anja Buchbinder, Rory Edward Morty, Britta Bausch, Tobias Fischer, Richard Schulz, Friedrich Grimminger, Martin Witzenrath, Matt Barnes, Werner Seeger, Konstantin Mayer

**Affiliations:** University of Giessen and Marburg Lung Center (UGMLC), Justus-Liebig-University of Giessen, Klinikstr. 33, Giessen, D – 35392 Germany; Department of Lung Development and Remodelling, Max Planck Institute for Heart and Lung Research, Bad Nauheim, Germany; Charité - Universitätsmedizin Berlin, Medizinische Klinik mit Schwerpunkt Infektiologie und Pneumologie, Berlin, Germany; Takeda Cambridge Ltd, Cambridge, UK; University of Giessen and Marburg Lung Center (UGMLC), Medical Clinic II, Klinikstr. 33, Giessen, 35392 Germany

## Abstract

**Introduction:**

Acute respiratory distress syndrome (ARDS) is a major cause of mortality in intensive care units. As there is rising evidence about immuno-modulatory effects of lipid emulsions required for parenteral nutrition of ARDS patients, we sought to investigate whether infusion of conventional soybean oil (SO)-based or fish oil (FO)-based lipid emulsions rich in either n-6 or n-3 fatty acids, respectively, may influence subsequent pulmonary inflammation.

**Methods:**

In a randomized controlled, single-blinded pilot study, forty-two volunteers received SO, FO, or normal saline for two days. Thereafter, volunteers inhaled pre-defined doses of lipopolysaccharide (LPS) followed by bronchoalveolar lavage (BAL) 8 or 24 h later. In the murine model of LPS-induced lung injury a possible involvement of resolvin E1 (RvE1) receptor ChemR23 was investigated. Wild-type and ChemR23 knockout mice were infused with both lipid emulsions and challenged with LPS intratracheally.

**Results:**

In volunteers receiving lipid emulsions, the fatty acid profile in the plasma and in isolated neutrophils and monocytes was significantly changed. Adhesion of isolated monocytes to endothelial cells was enhanced after infusion of SO and reduced by FO, however, no difference of infusion on an array of surface adhesion molecules was detected. In neutrophils and monocytes, LPS-elicited generation of pro-inflammatory cytokines increased in the SO and decreased in the FO group. LPS inhalation in volunteers evoked an increase in neutrophils in BAL fluids, which decreased faster in the FO group. While TNF-α in the BAL was increased in the SO group, IL-8 decreased faster in the FO group. In the murine model of lung injury, effects of FO similar to the volunteer group observed in wild-type mice were abrogated in ChemR23 knockout mice.

**Conclusions:**

After infusion of conventional lipid emulsions, leukocytes exhibited increased adhesive and pro-inflammatory features. In contrast, FO-based lipid emulsions reduced monocyte adhesion, decreased pro-inflammatory cytokines, and neutrophil recruitment into the alveolar space possibly mediated by ChemR23-signaling. Lipid emulsions thus exert differential effects in human volunteers and mice *in vivo*.

**Trial registration:**

DRKS00006131 at the German Clinical Trial Registry, 2014/05/14

**Electronic supplementary material:**

The online version of this article (doi:10.1186/s13054-015-0933-6) contains supplementary material, which is available to authorized users.

## Introduction

Acute respiratory distress syndrome (ARDS) is still linked with a high mortality rate. Despite major advances in intensive care medicine, a successful pharmacologic approach for the management of patients with ARDS is still missing [1]. Pro-inflammatory mediators have been suggested to contribute to primary and secondary organ dysfunction in experimental models of ARDS, and excessive generation of these mediators has been observed in patients [2].

Monocytes have been suggested to be intimately involved in controlling inflammatory cascades [3], as they release both pro-and anti-inflammatory cytokines directing activation and recruitment of leukocyte populations, such as polymorphonuclear granulocytes (PMN). The PMN represent the first line of defense against invading bacteria, yet they are capable of causing serious tissue destruction [4].

Eicosanoids play an essential role in the modulation of pro-inflammatory and anti-inflammatory events [5,6]. The n-6 fatty acids, including arachidonic acid, represent the predominant polyunsaturated fatty acid in common Western diets, and current nutritional regimes. Eicosapentaenoic acid and docosahexaenoic acid are the most important members of the n-3 family of fatty acids. Both may serve as alternative lipid precursors for the cyclooxygenase and lipoxygenase pathways [6]. Moreover, by incorporation into various membrane (phospho)-lipid pools, n-6 and n-3 fatty acids may affect lipid-signaling events and may be mediated by the potent pro-resolution lipid mediator class of resolvins [7,8].

Diets with specific fat composition may influence inflammatory and immunological events. Beneficial effects of n-3 fatty acids have been demonstrated in experimental models of acute lung injury [9,10]. In patients with lung injury or sepsis, an enteral diet enriched in n-3 fatty acids and anti-oxidants reduced ventilation time, improved the oxygenation index, and reduced the length of stay in the intensive care unit [11]. However, recent studies including a large multi-center trial conducted by the ARDSnet (OMEGA) investigating the effect of an enteral supplementation of n-3 fatty acids in ARDS patients revealed no beneficial effect [12,13]. The study OMEGA was stopped early for futility, displaying a higher rate of complications in the group receiving n-3 fatty acids. Due to the inconsistency of data on the enteral use of n-3 fatty acids in ARDS there is an ongoing debate in the scientific community with a final recommendation lacking at the moment. Data on the use of n-3-based lipid emulsions in parenteral nutrition in ARDS or even to reduce subsequent injury as currently applied are scarce [14,15].

In the present study, we assessed the impact of two commercially available lipid emulsions on isolated leukocyte function and intra-alveolar recruitment of leukocytes on subsequent endotoxin inhalation in healthy volunteers. As recent studies suggest an involvement of the novel n-3 derived lipid mediator resolvin E1 (RvE1) and its receptor ChemR23 in the mediation of n-3-induced (patho-)physiological effects, we additionally subjected wild-type and ChemR23 knockout mice to a model of experimental ARDS after infusion of lipid emulsions.

## Material and methods

### Study design

The University Ethics Committee (*Ethikkommission des Fachbereichs Medizin*, Justus-Liebig University Giessen) approved the study, and written informed consent was obtained from each volunteer. The study was registered as DRKS00006131 at the German Clinical Trial Registry. Forty-two volunteers were recruited and blood was drawn by venipuncture at 8 am of the first day. Via antecubital venous access, a heparin infusion with 10,000 units/day was started for 48 h. Volunteers were then randomized by closed envelopes into blocks of six to receive either 350 ml of a 10% fish oil (FO)-based lipid emulsion (Omegaven®), a 10% soybean oil (SO)-based lipid emulsion (Lipoven®), or NaCl 0.9% on both day 1 and day 2 (composition of the lipid emulsions is detailed in Additional file 1: Table E1). Each infusion was started at 4 pm and lasted for 12 h. At 4 h after completion of the second infusion (8 am on the third day), the volunteers inhaled the endotoxin lipopolysaccharide (LPS). After 8 or 24 h, blood was drawn by antecubital venipuncture and the volunteers underwent bronchoscopy with bronchoalveolar lavage. Both volunteers and investigators performing the laboratory investigations were blinded to the nature of lipid emulsion employed.

### Volunteer selection

The volunteers were >18 years of age, did not smoke, and were not vegetarians. They did not take FO capsules or any comparable nutritional supplementation. Additional details are provided in the online data supplement.

### LPS inhalation and bronchoalveolar lavage

The LPS inhalation was carried was carried out as described by Kline *et al*. [16]. Further details are provided in the online data supplement.

### Preparation of endothelial cells

Endothelial cells were obtained from human umbilical veins according to Jaffe *et al*. [17].

### Monocyte isolation

Human monocytes were isolated by density gradient centrifugation, followed by counterflow centrifugation elutriation [18].

### Preparation of human granulocytes

Neutrophils were isolated by density gradient centrifugation [19].

### Quantitative RT-PCR

Total RNA was extracted from freshly separated PMN, subsequently reverse-transcribed and analyzed by quantitative real-time PCR (PE Applied Biosystems, Wellesley, MA, USA).

### Monocyte adhesion and rolling assay

Monocyte adhesion and rolling were determined using a parallel plate flow chamber [18].

### Immunofluorescence analysis

Blood anticoagulated with EDTA was subjected to immunofluorescence [18]. Additional details are provided in the online data supplement.

### Culture and stimulation of monocytes and neutrophils

Monocytes or neutrophils (5 × 10^5^ in a 24-well tissue culture plate) were cultivated and stimulated with vehicle only (control) or 10 ng/ml LPS for 24 h at 37°C, 5% CO_2_. Measurement of cytokines (TNF-α, IL-1β, IL-8, IL-10) was performed in cell culture supernatant using commercially available ELISA Kits (R&D Systems, Wiesbaden, Germany) according to the manufacturer’s instructions.

### Analysis of fatty acids

Analysis of cellular lipids and free fatty acids was performed by means of gas chromatography [18].

### Animals and experimental lung injury protocol

Local government authorities and university officials responsible for animal protection approved the study (Justus-Liebig University Giessen, *Regierungspraesidium* Giessen). The murine model of long-term infusion and subsequent acute lung injury has been previously described [20]. Wild-type (Sv129/S1) and ChemR23−/− mice [21] were used for experiments. After implantation of a jugular vein catheter a subsequent adaptation to an osmotic mini-pump (Alzet, Cupertino, CA, USA) was performed. Seven days after central venous catheter implantation in mice, continuous infusion (6.5 μl/h) of either 10% FO-based lipid emulsion (Omegaven®, Fresenius Kabi, Bad Homburg, Germany), 10% SO-based lipid emulsion (Lipoven®, Fresenius Kabi, Bad Homburg, Germany), or NaCl 0.9% was performed with the mice being allowed access to water and chow *ad libitum*. The amount of lipids infused is equivalent to 1.0 g/kg/d. However, energy expenditure of mice is nearly three times higher compared to humans. Therefore, the infused lipids were considered to be close to the lower limits of the recommended amount of lipids in parenteral nutrition. While receiving infusions, mice were subcutaneously injected with a low dose of unfractionated heparin. Thereafter, anesthetized mice were instilled with LPS (0 or 1 μg in 200 μl normal saline/mouse): 8 or 24 h after LPS application, mice were sacrificed by an overdose of anesthesia, and bronchoalveolar lavage was performed.

### Statistical analysis

Data are provided as the mean ± standard error of the mean (SEM). Two-way analysis of variance (ANOVA) was used to test for differences between time points (baseline, 8 h, and 24 h) and infusion groups (NaCl, SO, FO). *Post hoc* analysis was carried out using the Student-Newman-Keul test. If data were not normally distributed, logarithmic transformation was performed. In the case of bronchoalveolar lavage, two-way ANOVA across time points (8 and 24 h) and between infusion groups (NaCl, SO, FO) was used for comparison. A *P*-value <0.05 was considered to indicate statistical significance.

## Results

### Clinical course

All volunteers but one received a complete infusion course of FO-based lipid emulsions, SO-based lipid emulsions, or NaCl in a randomized fashion (Additional file 1: Figure E1). During the infusion periods, no adverse events occurred. All infusions were well-tolerated, no overt bleeding was noted, and the volunteers did not report problems concerning with the infusion site. After LPS inhalation, most volunteers reported chill, fatigue, and coughing but the symptoms resolved within 6 h.

### Leukocyte invasion after LPS-inhalation

**Figure 1:**
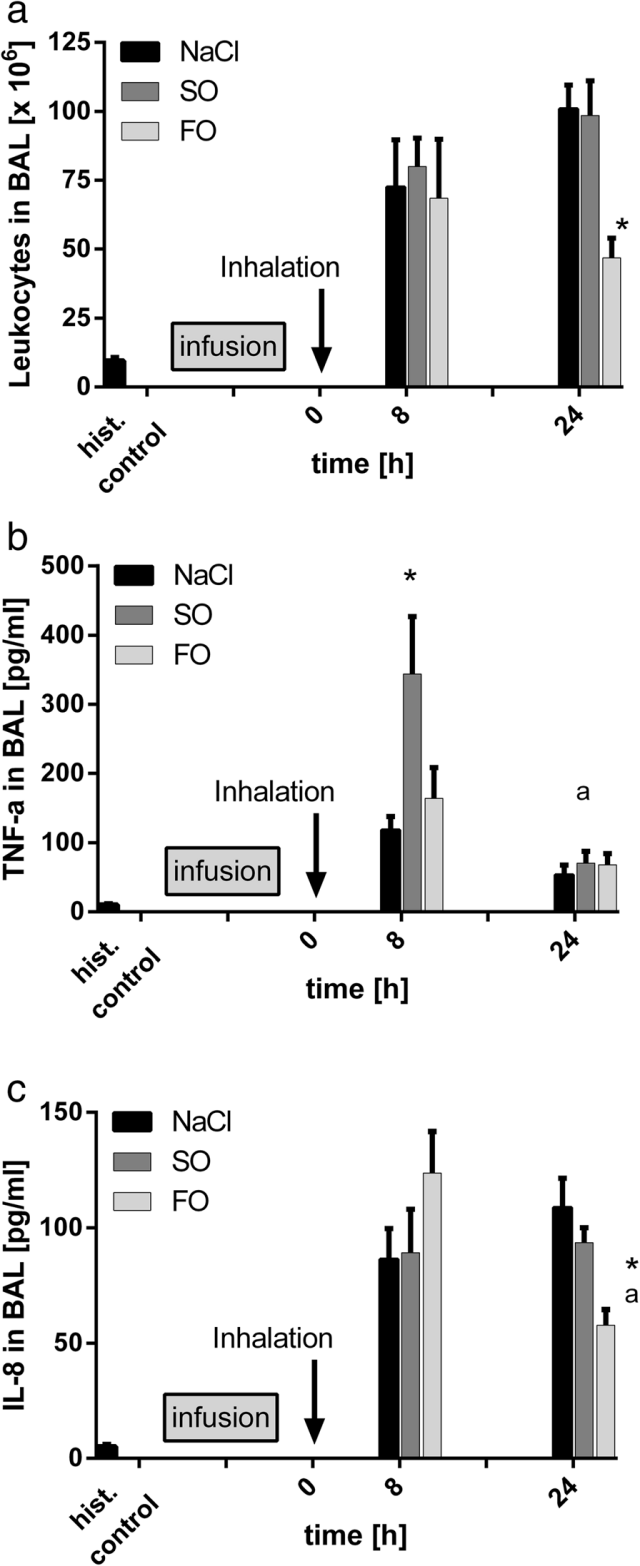
Impact of infusions on leukocytes and cytokines in the bronchiolar lavage fluid (BALF) after lipopolysaccharide (LPS)-inhalation. BAL was performed 8 or 24 h after LPS inhalation in volunteers undergoing infusion of soybean-based lipid emulsions (SO), fish oil-based lipid emulsions (FO), or normal saline (NaCl). For comparison, a cohort of healthy volunteers (historical controls) is depicted. Total leukocytes **(a)**, TNF-α **(b)**, and IL-8 **(c)** were determined in the BALF: 24 h after LPS challenge lower leukocyte numbers were detected in the FO group (^*^ *P* <0.05 versus both other groups). TNF-α was increased in the SO group at 8 h but was similar to the other infusion groups at 24 h (^*^ *P* <0.05 versus both other groups; ^a^ *P* <0.05 versus 24 h). Lowest IL-8 concentrations were determined in the FO group at 24 h (^*^ *P* <0.05 versus both other groups; ^a^ *P* <0.05 versus 8 h). Data are given as mean +/− standard error of the mean; n = 5 to 6 experiments each.

Recovery of the 150-ml normal saline instilled for lavage did not differ significantly between the groups. In a historical control group of healthy volunteers, we found 9.5 ± 1.3 × 10^6^ leukocytes and 0.3 ± 0.1 × 10^6^ PMN in bronchoalveolar lavage fluid (BALF). In volunteers receiving NaCl-infusions, 72.6 ± 17.2 × 10^6^ leukocytes were detected in the BALF 8 h after LPS inhalation with a predominant neutrophil population of 35.7 ± 8.3 × 10^6^ cells (Figure 1a). Macrophages and monocytes accounted for 9.1 ± 2.7% and lymphocytes for 41.3 ± 4.2% of the leukocytes (Additional file 1: Figure E2). Leukocytes further increased 24 h after inhalation showing a neutrophil predominance again. Infusion of SO-based lipid emulsions induced leukocyte counts in BALF similar to those in the NaCl group 8 and 24 h after LPS inhalation. Volunteers receiving FO-based lipid emulsions displayed similar leukocyte counts compared to the other groups 8 h after LPS-inhalation. A key difference in the FO group was a decrease in leukocytes in BALF after 24 h, as leukocytes and neutrophils differed significantly from both other groups at this time point (*P* <0.05). The leukocyte differentiation pattern did not differ significantly between the groups at either time point.

### TNF-α and IL-8 in bronchoalveolar lavage fluid (BALF)

We then determined cytokines in the BALF to gain insight into inflammatory activation after LPS inhalation. In our historical control group of healthy volunteers, concentrations of TNF-α and IL-8 were close to the detection limit. In the NaCl-group, TNF-α concentration in BALF (Figure 1b) increased 8 and 24 h after LPS-inhalation, respectively, and was comparable to the concentrations determined in the group receiving FO-based lipid emulsions. After infusion of SO-based lipid emulsions, TNF-α concentrations were more markedly increased in BALF 8 h after LPS inhalation, and differed significantly from both other groups (*P* <0.05). Levels of TNF-α in BALF were lower in all groups at 24 h compared to their respective 8 h concentrations but the decrease was only significant for the n-6 group (*P* <0.05).

The IL-8 concentrations increased in the NaCl group 8 h after LPS inhalation and remained elevated at 24 h (Figure 1c). While IL-8 concentrations were nearly identical in volunteers receiving SO-based lipid emulsions, they were 43% higher in the FO group at 8 h compared to the NaCl group and significantly lower at 24 h, differing at this time point from both other groups (*P* <0.05).

### IL-8 generation in isolated neutrophils

**Figure 2:**
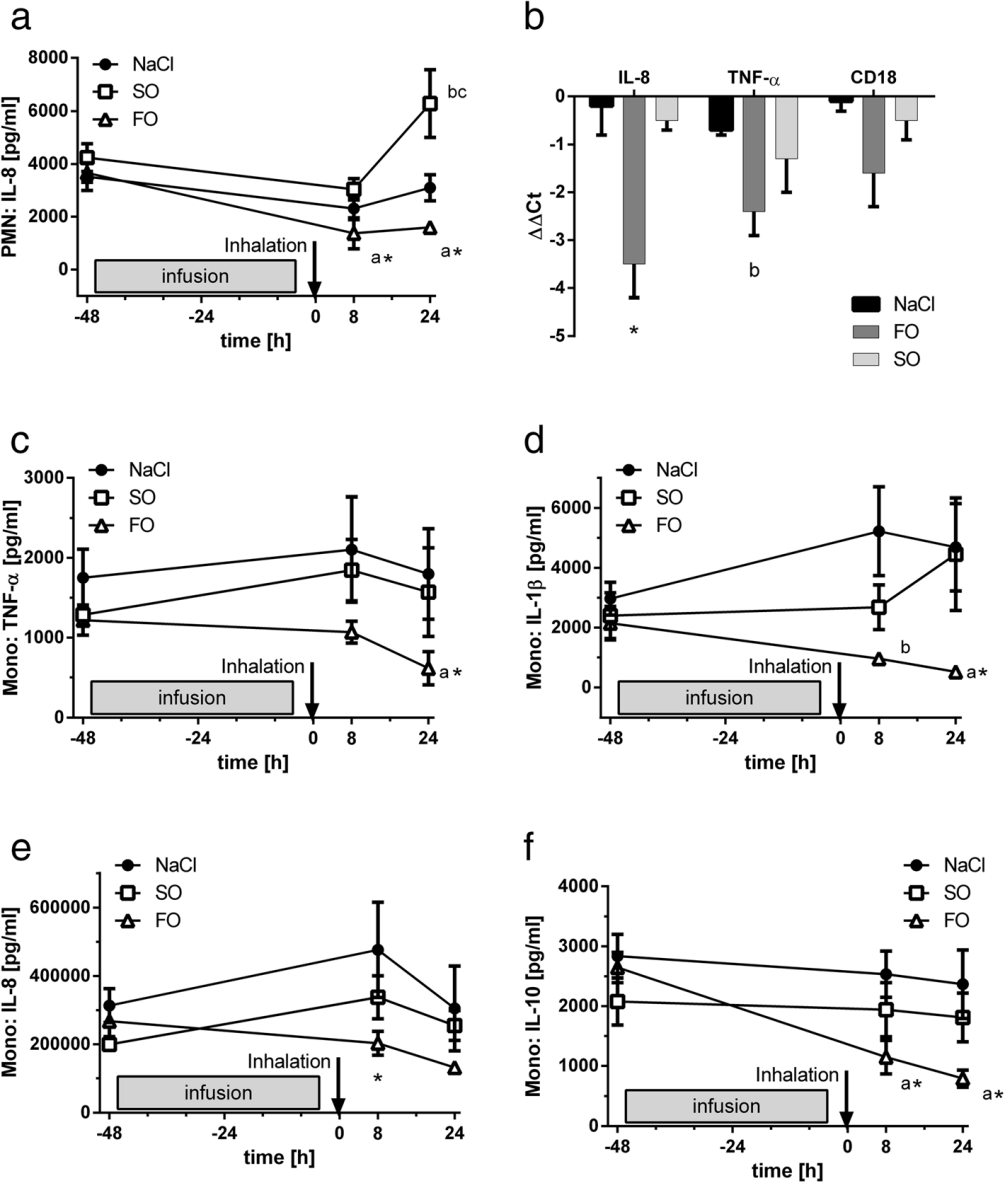
Impact of infusions on leukocyte cytokine release. Polymorphonuclear cells (PMN) **(a, b)** or monocytes **(c-f)** originated from volunteers receiving fish oil-based (FO) or soybean oil-based (SO) lipid emulsions, or normal saline (NaCl). RNA was extracted from PMN **(b)**; quantitative PCR was performed and ΔΔCt values were calculated (see methods). Leukocytes were stimulated with lipopolysaccharide (LPS) and cytokine release was assessed after 24 h. Expression **(b)** of IL-8 and TNF-α were reduced in the FO group as compared to the SO and NaCl (*P <0.05) and NaCl groups (^a^ *P* <0.05), respectively). Generation of IL-8 by PMN **(a)** was increased in the SO group but reduced in the FO group (*P <0.05 versus baseline; ^a^ *P* <0.05 versus SO and NaCl; ^b^ *P* <0.05 versus 8 h; ^c^ *P* <0.05 versus NaCl). TNF-α in monocytes **(c)** was reduced in the FO group (*P <0.05 versus baseline and 8 h; ^b^ *P* <0.05 versus SO and NaCl). IL-1 **(d)** also decreased in the FO group (^b^ *P* <0.05 versus NaCl; ^a^ *P* <0.05 versus NaCl and SO; *P <0.05 versus baseline). In contrast, IL-8 synthesis **(e)** in the FO group did only differ at 8 h from the NaCl group (*P <0.05). IL-10 **(f)** was decreased in a similar manner in the FO group (*P <0.05 versus baseline; ^a^ *P* <0.05 versus NaCl and SO). Data are given as mean ± standard error of the mean; n = 12 (baseline) or 5 to 6 (post-inhalation) experiments each. Error bars are not evident when obscured by the symbol.

As the next step, we investigated the effect of lipid emulsion and LPS inhalation on the cytokine release of PMN isolated from blood. Before LPS inhalation and infusion, IL-8 generation in neutrophils elicited by 10 ng/ml LPS was 3,515 ± 530 pg/ml in the NaCl group (Figure 2a). The IL-8 secretion decreased to 66% at 8 h and to 88% at 24 h after LPS inhalation in this group. In the FO group, IL-8 synthesis decreased significantly from 3,659 ± 353 pg/ml before inhalation to 38% and 44%, at 8 and 24 h, respectively, after LPS inhalation (*P* <0.05, 8 and 24 h versus baseline). In the SO group, generation of IL-8 also decreased to 72% 8 h after LPS inhalation. In contrast to the NaCl and the FO groups, IL-8 synthesis increased to 6,277 ± 1276 pg/ml at 24 h in the SO group (*P* <0.05 versus FO and versus baseline).

### TNF-α, CD18 and IL-8 gene expression in PMN

To assess the gene expression of TNF-α, CD18 and IL-8, PMN were isolated from the blood 24 h after LPS inhalation and subjected to quantitative RT-PCR (Figure 2b). The ΔΔCt values were calculated comparing gene expression (normalized to a housekeeping gene) of the different treatment groups to their corresponding values before treatment. In the group receiving FO-based lipids, PMN exhibited a significantly reduced expression of IL-8 compared to volunteers receiving SO-based lipid emulsions and the NaCl group (*P* <0.05). The TNF-α expression was furthermore decreased in the FO group (*P* <0.05), whereas the group infused with SO-based lipid emulsion did not reduce TNF-α levels significantly. Expression of CD18 did not differ significantly among the groups tested.

### Generation of TNF-α, IL-1β, IL-8, and IL-10 in isolated monocytes

Before LPS inhalation and infusion, generation of TNF-α in isolated monocytes stimulated with 10 ng/ml LPS was 1,752 ± 359 pg/ml in the group receiving NaCl and did not differ significantly between infusion groups (Figure 2c). In the groups infused with NaCl and SO TNF-α synthesis increased 8 h after LPS inhalation and returned to pre-inhalation levels after 24 h. In volunteers receiving FO-based lipid emulsions, TNF-α synthesis was significantly reduced 24 h after LPS inhalation (*P* <0.05; 24 h versus baseline).

Baseline synthesis of IL-1β in isolated monocytes challenged with 10 ng/ml LPS in the control group was 2,973 ± 548 pg/ml (Figure 2d) increasing 8 h and 24 h after LPS inhalation. In the SO group, IL-1β synthesis exhibited a similar pattern, whereas in the group receiving FO-based lipid emulsions, IL-1β synthesis decreased 8 h and 24 h after inhalative LPS challenge. The IL-1β generation 24 h after LPS-challenge in the FO group differed significantly from its baseline and from the NaCl group (*P* <0.05 for each comparison).

The baseline LPS-induced generation of IL-8 was 3,13.1 ± 50.0 ng/ml in the NaCl group and neither of the other groups differed significantly at this time point (Figure 2e). The IL-8 synthesis peaked 8 h after LPS inhalation and returned to baseline after 24 h in the NaCl group but the time points did not differ significantly from baseline. While the SO group displayed a similar pattern, a consistent reduction in IL-8 generation after LPS inhalation in the FO group was observed, which differed significantly from the NaCl group at 8 h (*P* < 0.05).

Last, the LPS-induced synthesis of the anti-inflammatory cytokine IL-10 in isolated monocytes was examined (Figure 2f). Baseline generation determined in the NaCl group was 2,838 ± 364 pg/ml and the volunteers in both lipid groups displayed similar values. The IL-10 synthesis was not significantly affected by LPS inhalation and infusion in the NaCl and SO groups. In contrast, after infusion of FO-based lipid emulsions and LPS-challenge we found a significant reduction of IL-10 generation (*P* <0.05, FO versus both other groups and baseline versus both post-inhalation time points).

### Adhesion and rolling

**Figure 3:**
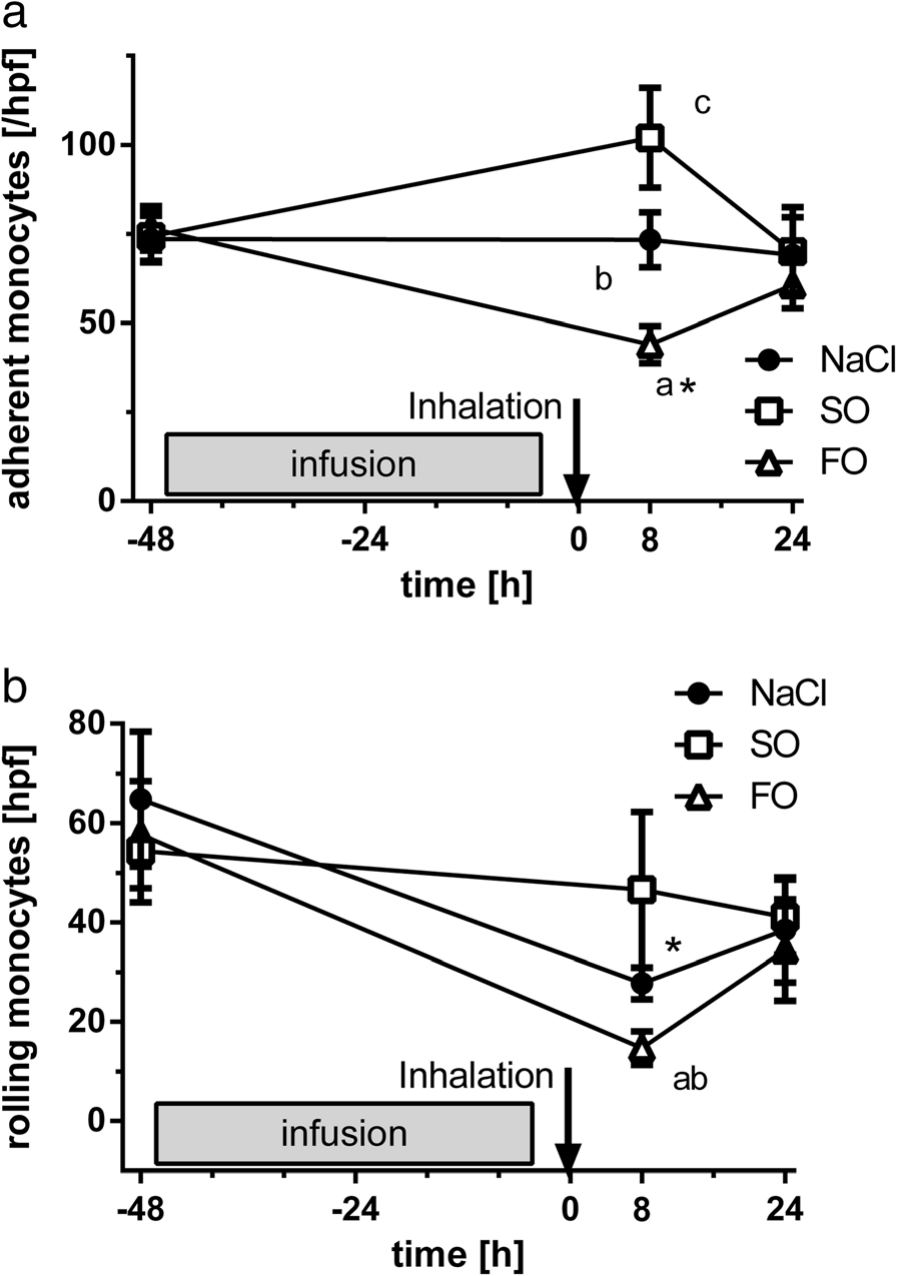
Impact of infusions on monocyte rolling and adhesion to a human endothelial monolayer after LPS inhalation. Isolated monocytes originated from volunteers receiving fish oil (FO)- or soybean oil (SO)-based lipid infusion, or normal saline (NaCl). Adhesion **(a)** and rolling **(b)** were investigated on TNF-α-activated endothelial cells under laminar flow conditions (mean ± standard error of the mean; n = 12 for baseline and 5 to 6 for post-inhalation experiments). Adhesion was significantly different 8 h after inhalation between all groups (^a^FO versus SO, *P* <0.01; ^b^NaCl versus FO or SO, *P* <0.05. The increased adhesion in the SO group was significantly different from its baseline and 24-h values (^c^ *P* <0.05 for each comparison). At 8 h post-inhalation, adhesion in the FO group was lower as compared to its baseline (^*^ *P* <0.05). Rolling was reduced in all infusion groups 8 h after LPS inhalation and the reduction was significant in the NaCl and FO groups (^a^ *P* <0.05; ^*^ *P* <0.01 versus respective baseline and *P* <0.05 versus 24 h). Rolling at 8 h was lower in monocytes isolated after FO infusion as compared to both other groups (^b^ *P* <0.01 versus SO and *P* <0.05 versus NaCl).

We then determined if lipid emulsions and LPS inhalation changed the adhesive properties of monocytes using a parallel flow chamber (Figure 3). Before infusion and inhalation, 73.6 ± 6.6 monocytes were adherent in the NaCl group, a feature unchanged at 8 and 24 h after inhalation. Eight hours after LPS inhalation, the number of adherent monocytes was reduced in the FO group but increased in the SO group. All groups differed significantly at this time point (*P* <0.05). After 24 h, the number of adherent monocytes reached pre-infusion values in all groups. The number of adherent monocytes in the FO group at 8 h differed significantly from its baseline while the SO group at 8 h was significantly different from both other time points.

Under baseline conditions, similar numbers of monocytes were rolling in the NaCl, FO, and SO group. Eight hours after infusion and LPS inhalation, the number of rolling monocytes dropped to 46% in the NaCl group but decreased only slightly in the SO group. The number of rolling monocytes were significantly reduced to 25% in the FO group (*P* <0.05, baseline versus 8 h post-inhalation) differing significantly from both other groups (*P* <0.05). At 24 h after inhalation, rolling monocytes increased in the NaCl and FO groups (*P* <0.05 versus 8 h).

### Flow-cytometric analysis in monocytes and PMN

Next we sought to examine if the changes in rolling and adhesion were mirrored in alterations of surface adhesion molecules. Monocytes and PMN were examined for changes in expression of adhesion molecules CD11b (Mac-2), CD14, CD18, CD45, CD49d (VLA-4), CD62L (L-selecin), CD162 (P-selectin glycosylated ligand (PSGL)-1), and CC-chemokine receptor 2 (CCR2) and CCR5 by flow cytometry (Additional file 1: Table E2 + E3). Two-way ANOVA of monocytes revealed time-dependent effects if all groups were pooled, which became significant only in part in the individual treatment groups. The CD11b exhibited a significant time-dependent response, with expression peaking at 8 h (*P* <0.01) but was only significant in the FO and SO group. A similar pattern was observed for CD18, which was upregulated after 8 h in all groups. Only the FO group was significantly different from baseline. Expression of CD49d in the pooled groups was decreased at 8 h, and significantly increased values at 24 h, but only the SO group differed significantly at 24 h compared to the 8-h values. Interestingly, we could not reveal any inter-group difference for any surface molecules examined despite significant changes in the adhesive properties in the cell assay.

In neutrophils, a significantly lower expression of CD11b 24 h after LPS challenge (*P* <0.05 versus baseline and versus 8 h) was detected in all infusion groups. The CD14 exhibited increased expression in all groups, both 8 and 24 h after LPS stimulation, compared to baseline. Expression of all the other markers tested displayed no significant changes comparing pre-infusion to post-inhalation values within one infusion group. In addition, no significant differences were found between infusion groups.

### Plasma free fatty acids

The sum of all free fatty acids in plasma in the NaCl group before the start of infusions was 335.1 ± 50.2 μmol/l (Table 1). Levels did not change significantly 8 or 24 h after inhalation with both other groups exhibiting the same pattern. Free arachidonic acid levels were determined as 4.0 ± 0.6 μmol/l at baseline in the NaCl group (Table 1). No significant differences in levels were determined between the different groups and after inhalation with each group. For both n-3 fatty acids, no significant differences in fatty acid concentrations were found at baseline between the infusion groups (Table 1). While concentrations in the NaCl and SO groups remained stable, eicosapentaenoic acid levels rose in the group receiving FO-based emulsions 8 h and 24 h after LPS inhalation. An increase was also found in levels of docosahexaenoic acid, peaking at 24 h. At 8 and 24 h, both fatty acids differed significantly from baseline and from concentrations in the NaCl and SO group (*P* <0.05 for each comparison.

**Table 1:** **Fatty acids in the plasma and monocyte membranes**

		**Time, hours**		
	**Group**	**0**	**8**	**24**
**Free fatty acid, μmol/l**				
AA	NaCl	4.0 ± 0.6	3.3 ± 0.3	4.9 ± 1.0
	SO	4.0 ± 0.6	4.0 ± 0.7	4.6 ± 1.3
	FO	4.3 ± 0.5	4.2 ± 0.8	5.0 ± 1.1
EPA	NaCl	0.6 ± 0.1	0.3 ± 0.0	0.4 ± 0.1
	SO	0.6 ± 0.1	0.5 ± 0.1	0.7 ± 0.2
	FO	0.7 ± 0.2	3.4 ± 0.8^ab^	2.5 ± 0.4^ab^
DHA	NaCl	0.8 ± 0.1	0.6± 0.2	0.9 ± 0.2
	SO	0.8 ± 0.1	0.9 ± 0.1	1.0 ± 0.2
	FO	0.9 ± 0.1	2.2 ± 0.3^a b^	3.0 ± 0.7^a b^
Sum	NaCl	335.1 ± 50.2	336.8 ± 42.4	408.6 ± 74.3
	SO	346.0 ± 53.9	372.1 ± 42.1	366.5 ± 59.8
	FO	377.4 ± 44.1	250.1 ± 29.8	348.3 ± 83.0
**Membrane fatty acid. %**				
AA	NaCl	19.7 ± 0.5	19.2 ± 0.6	18.5 ± 0.5
	SO	19.4 ± 0.4	18.0 ± 0.8	20.0 ± 0.3
	FO	19.4 ± 0.6	17.1 ± 0.8^a^	17.4 ± 1.1^a^
EPA	NaCl	0.2 ± 0.0	0.1 ± 0.0	0.1 ± 0.0
	SO	0.2 ± 0.0	0.1 ± 0.0	0.2 ± 0.0
	FO	0.2 ± 0.0	1.9 ± 0.2^a b^	1.3 ± 0.1^a b^
DHA	NaCl	2.6 ± 0.1	2.4 ± 0.3	2.3 ± 0.2
	SO	2.9 ± 0.2	2.5 ± 0.3	2.8 ± 0.4
	FO	3.1 ± 0.2	4.8 ± 0.2^a b^	4.7 ± 0.4^a b^

Plasma and isolated monocytes originated from volunteers receiving fish oil (FO)- or soybean oil (SO)-based lipid infusion, or normal saline (NaCl). Free fatty acids (AA, arachidonic acid; EPA, eicosapentaenoic acid; DHA, docosahexaenoic acid) and membrane fatty acids were determined by gas chromatography: ^a^ *P* <0.05 versus baseline; ^b^ *P* <0.05 versus SO and NaCl. Data are presented as mean +/- standard error of the mean; n =12 for baseline and n = 5 – 6 for later time points.

### Membrane fatty acid profile in monocytes

As fatty acid composition of the cell membrane may influence intracellular signal transduction we determined the fatty acid profile of monocytes isolated from blood. Baseline arachidonic acid content in monocytes was 19.70 ± 0.45% of all fatty acids in the NaCl group and was slightly reduced after LPS inhalation (Table 1). In the FO group, arachidonic acid content was reduced, and differed significantly from baseline (*P* <0.05 at 8 h and 24 h versus baseline). In the FO group, eicosapentaenoic acid and docosahexaenoic acid increased markedly 8 h and 24 h, respectively, after LPS inhalation (Table 1). Levels of both fatty acids differed significantly from baseline values, and from the other infusion groups at these time points. The ratio of arachidonic acid: (eicosapentaenoic acid + docosahexaenoic acid) did not significantly change in the NaCl and SO groups. However, it was 6.4:1 at baseline, and was decreased at 8 or 24 h in the n-3 group (2.6: 1 and 2.9:1; *P* <0.05 versus baseline and between groups).

### Membrane fatty acid profile in neutrophils

A similar pattern compared to the free fatty acids was detected in the membrane lipids of PMN (Additional file 1: Figure E3). Baseline arachidonic acid content was 9.00 ± 0.20% of all fatty acids in the NaCl group, and was similar in all groups and all time points. In the FO group, eicosapentaenoic acid increased about three to four times 8 h and 24 h after LPS inhalation. Docosahexaenoic acid levels rose from 0.55 ± 0.05% to 0.75 ± 0.11% and 0.79 ± 0.10%. Both fatty acids differed significantly from baseline and between the groups at these time points. The ratio of arachidonic acid:(eicosapentaenoic acid + docosahexaenoic acid) did not change in the NaCl and SO groups. However, this ratio was 14.5:1 at baseline, and dropped significantly at 8 or 24 h in the FO group (6.5:1 and 7.1:1; *P* <0.05 versus baseline and between groups).

### Chemerin receptor 23 (ChemR23) in experimental acute lung injury

**Figure 4:**
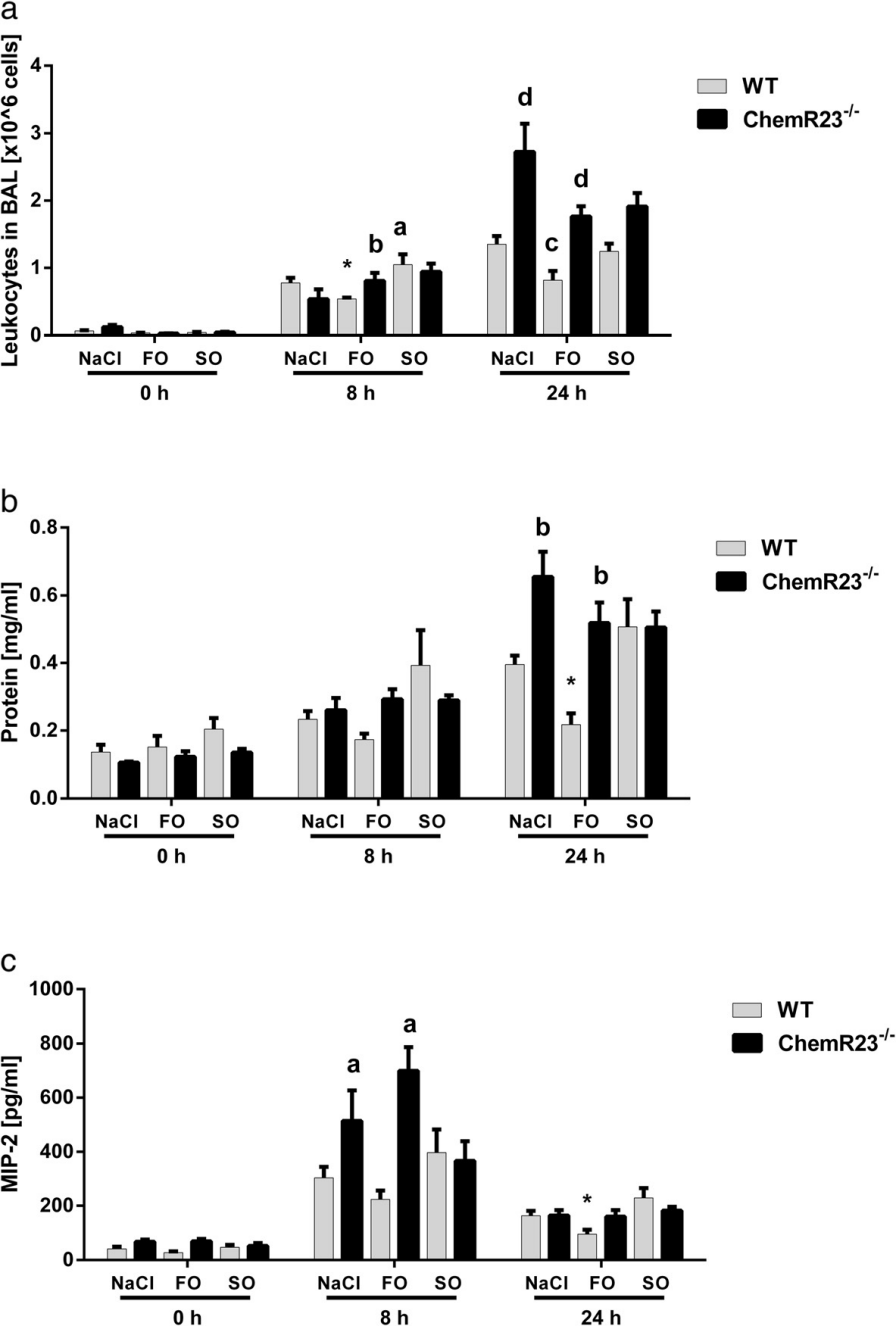
Chemerin receptor 23 (ChemR23) in experimental acute lung injury (ALI). Wild-type (WT) and ChemR23 knockout (ChemR23−/−) mice received infusions with NaCl, fish-oil (FO)- or soybean oil (SO)-based lipid emulsions and were subjected to lipopolysaccharide (LPS) for the indicated time points. **(a)** WT mice receiving FO displayed the lowest leukocytes in their bronchiolar lavage fluid (BALF) at 8 h compared to NaCl (*P <0.05 versus NaCl), whereas the SO group had the highest values (^a^ *P* <0.05 versus FO and NaCl). The effect of FO-treatment was diminished in ChemR23−/− showing significantly increased alveolar leukocyte invasion compared to WT (^b^ *P* <0.05). After 24 h, the WT-FO group displayed lowest leukocytes counts (^c^ *P* <0.05 versus NaCl and SO). At this time point, ChemR23−/− mice of the NaCl and FO group revealed significantly elevated alveolar leukocytes compared to the respective WT (^d^ *P* <0.05). **(b)** Protein extravasation 24 h after LPS challenge in WT animals infused with FO was lowest compared to NaCl and SO (*P <0.05), whereas ChemR23−/− mice showed significantly increased protein leakage in mice infused with NaCl or FO compared to the respective WT controls (^b^ *P* <0.05). **(c)** Macrophage inflammatory protein (MIP)-2 levels 8 h after LPS challenge were significantly elevated in ChemR23−/− mice infused with NaCl or FO compared with the respective WT animals (^a^ *P* <0.05). After 24 h the lowest MIP-2 levels were detected in the FO group of WT mice compared to NaCl and SO (*P <0.05).

To elucidate potential underlying mechanisms involved in the beneficial role of FO in ARDS we used the murine model of LPS-induced acute lung injury (ALI). LPS-instillation increased alveolar recruitment of leukocytes 8 and 24 h after induction of ALI in wild-type (WT) animals (Figure 4a). After 8 h, FO-infused WT mice displayed a significant decrease in alveolar leukocyte counts as compared to NaCl-treated animals (*P* <0.05), whereas SO-infused mice displayed the highest values compared to FO and NaCl (*P* <0.05). The beneficial effect of FO infusion was diminished in ChemR23−/− as these animals showed significantly increased alveolar leukocyte invasion compared to WT (*P* <0.05). A similar pattern was observed 24 h after LPS instillation as mice infused with FO displayed the lowest leukocytes counts compared to NaCl and SO (*P* <0.05). Also at this time-point, ChemR23−/− mice of the NaCl and FO group revealed significantly elevated alveolar leukocytes compared to the respective WT groups (*P* <0.05).

Next, we investigated LPS-induced protein extravasation in ALI (Figure 4b). Despite an increase in protein concentration both at baseline (0 h) and after 8 h no significant changes occurred among the different groups. At 24 h after LPS challenge, WT animals infused with FO exhibited the lowest protein levels in the BALF compared to NaCl and SO (*P* <0.05), whereas this effect was abrogated in ChemR23−/− mice. In addition, we observed significantly increased protein leakage in ChemR23−/− mice infused with NaCl or FO compared to the respective WT controls (*P* <0.05). Finally, we sought to assess the concentration of the pro-inflammatory cytokine, macrophage inflammatory protein (MIP)-2 in BALF (Figure 4c). Consistent with the above mentioned results, MIP-2 levels 8 h after LPS challenge were significantly elevated in ChemR23−/− mice infused with NaCl or FO compared with the respective WT animals (*P* <0.05). After 24 h, the lowest MIP-2 levels were detected in the FO group of WT mice compared to NaCl and SO, whereas this observation was diminished in ChemR23−/− animals (*P* <0.05).

## Discussion

In the present study, a distinct and differential impact of SO- versus FO-based lipid emulsions on key leukocyte features was detected in healthy volunteers after an infusion period of 48 h and subsequent LPS inhalation. The fish oil (FO)-based lipid preparation shifted the n-3/n-6 ratio of plasma free fatty acids from an n-6 predominance to an n-3/n-6 balance, reduced *ex vivo* leukocyte pro-inflammatory cytokine generation, decreased isolated monocyte rolling and adhesion to endothelial cells, and decreased the number of PMN recruited into the bronchoalveolar compartment. In contrast, monocyte adhesion, neutrophil cytokine generation, and tumor-necrosis factor-α concentration in BALF were markedly enhanced by the standard SO-based lipid emulsion. These observed effects of FO-based lipid emulsions might at least in part be mediated by resolvin receptor ChemR23.

### Invasion of leukocytes into the alveolar space

Neutrophil invasion into the alveolar space after LPS challenge is a well-documented response in mice and men [16,22]. In mice, sequestration of neutrophils in the pulmonary capillaries takes place within one hour, trans-endothelial migration reaches a plateau between 12 and 24 h, and trans-epithelial migration peaks after 24 h [22]. In all volunteers, a massive increase in leukocytes and in particular in neutrophils in the BALF 8 h after LPS inhalation was noted. The number of leukocytes (70 to 80 million) needs to be compared to 9.5 × 10^6^ leukocytes found in BALF from a historical cohort of volunteers at our institution who were not exposed to LPS [23]. In volunteers receiving NaCl or SO-based lipid emulsions, leukocytes and neutrophils were even more increased after 24 h, which is well in line with kinetics in the murine model. In contrast, in volunteers with pre-infusion of FO-based lipids leukocytes and neutrophils were significantly decreased 24 h after LPS inhalation. This may be interpreted as reduced or shortened influx of neutrophils invading the alveolar space due to reduced cytokine generation and decreased adhesive properties. It is tempting to speculate that FO-based lipids shortened the time-frame for transmigration of neutrophils. Alternatively, resolvins, which are exclusively metabolized from eicosapentaenoic acid or docosahexaenoic acid, were shown to lead to a faster resolution of the allergic airway inflammation and may influence leukocyte kinetics [24]. Generation of these novel mediators may have fastened the resolution of the LPS-induced inflammation and increased the resolution of inflammatory response.

### Rolling and adhesion of monocytes

Monocytes adhere spontaneously to endothelial cell monolayers using a static assay, but substantial monocyte-endothelial adhesion under flow conditions demands preceding cytokine stimulation of the endothelial cells: E-selectin-L-selectin, ICAM-1-β_2_ integrin and in particular VCAM-1-VLA-4- interactions were shown to represent predominant adhesive forces under these conditions [25]. The finding of increased adhesive features after application of SO and reduced adhesion and rolling after infusion of FO-based lipids is corroborated by our previous study showing a similar effect of lipid emulsions in volunteers without LPS inhalation [18]. The marked change of endothelial adhesion and rolling of monocytes isolated after infusion of lipid emulsions might be mirrored in monocyte adhesion molecules. However, no major differences in expression of adhesion molecules between infusion groups were noted. This confirms previous data obtained from healthy volunteers undergoing infusion of lipid emulsions without LPS challenge [18]. However, the findings are in contrast to the observation that supplementation of the diet of healthy volunteers with 3 g FO per day for three weeks resulted in a significant reduction in the expression of ICAM-1 and CD11a on both freshly prepared and interferon-stimulated peripheral blood monocytes [26]. Interestingly, an effect of LPS inhalation on the upregulation of CC11b and CD18 was detected 8 h after LPS inhalation in all groups irrespective of infusion regimen. This upregulation contrasts with the finding of a reduced expression of CD11b in monocytes after LPS inhalation in asthmatic subjects [27] but differences in timing of the analysis and dose of inhaled LPS may account for the difference.

Irrespective of the common trend of increased expression of CD11b and CD18 in all groups 8 h after inhalation, rolling and adhesion differed between the infusion groups. It is speculated that changes in membrane lipid composition as mirrored by the n-3: n-6-ratio may be at least in part responsible for the difference. As rolling and adhesion of monocytes require cross-talk between the activated endothelium and monocytes, reduced intracellular and intercellular signal transduction after infusion of FO-derived lipids may translate into a decrease in adhesive properties of monocytes. Second messenger pathways include generation of inositol phosphates with a known reduction of these products in leukocytes after ingestion of fish oil capsules [28]. However, generation of inositolphosphates is only one part of a major network of signal transduction pathways (for example, phosphatidylinositol-3 kinase, diacylglycerol, membrane rafts, and phospholipase A_2_) being dependent on membrane lipids and modulated by their change [7,29]. These pathways and their actions are referred to as lipid signaling. A change in these lipid-dependent pathways may also account for a possible impact of SO- and FO-derived lipids on a change in avidity of integrins. However, it cannot be fully excluded that further adhesion molecules are responsible for the difference in rolling and adhesion of monocytes, such as chemokines of the GRO family [30], which were not determined in the present study.

### Changes in fatty acid profile

A rapid and sustained increase in free eicosapentaenoic acid and docosahexaenoic acid concentration was noted after infusion of FO-based lipids, counterbalancing arachidonic acid. The arachidonic acid:(eicosapentaenoinc acid + docosahexaenoic acid) quotient in this compartment shifted from n-6 predominance to an n-3:n-6-equivalence within 48 h of infusion therapy. This prompt appearance of free n-3 fatty acids indicated rapid hydrolysis of n-3 fatty acid-containing triglycerides supplied by the lipid emulsion. The rapid rate of appearance of free n-3 fatty acids exceeds corresponding alterations in response to conventional dietary FO uptake by order of magnitude [31]. The changes in plasma fatty acid profile are paralleled by an increase in n-3 fatty acids in the membranes of leukocytes. In fact, a significant increase in the n-3:n-6 fatty acid ratio in the monocyte and neutrophil membrane lipid pool was noted; however, within the short infusion time and high percentage of arachidonic acid in the membranes the changes were not as dramatic as in the plasma free fatty acid fraction.

### Cytokine generation in isolated monocytes and neutrophils

No significant difference in cytokine generation of the isolated blood leukocytes before and after LPS inhalation in the NaCl group was detected. It is possible that the absence of an LPS effect on circulating leukocytes is due to largely compartmentalized inflammation in the alveolar space. However, this idea is not supported by the upregulation of CD11b and CD18 on monocytes after LPS inhalation. On the other side, leukocytes activated due to the LPS challenge may sequester in the lung capillaries and transmigrate. The remaining circulating leukocytes assessed for this study may be a non-activated population.

The rate of generation of the pro-inflammatory cytokines TNF-α, IL-1, and IL-8 in monocytes and IL-8 in neutrophils induced by LPS was significantly reduced after infusion of FO-based lipid emulsions. In contrast, increased synthesis in IL-8 in neutrophils was noted in the SO group. A reduction in the generation of TNF-α after long-term dietary intake of FO capsules was first described by Endres *et al*. [32] and confirmed after infusion of FO-derived lipids [18,33]. However, the underlying mechanisms are not fully understood. As discussed for the adhesive properties, enrichment of membrane n-3 fatty acids may have a modulating effect on second messenger pathways. In addition, generation of eicosapentaenoic acid-derived eicosanoids such as leukotriene (LT)B_5_ and thromboxane (Tx)A_3_ has been demonstrated after infusion of FO-based lipid emulsions [34]. Such a shift might interfere with eicosanoid-dependent auto-amplification loops, as described for the regulation of TNF-α-synthesis in monocytes by TxA_2_ [35].

### Inflammatory cytokines in bronchoalveolar lavage fluid

Several groups have used bronchoscopy in healthy volunteers to investigate the impact of inhalation of LPS or LPS challenge on local inflammatory responses in the alveolar compartment, which is mirrored by invasion of neutrophils, and an increase in pro-inflammatory cytokines in the BALF, [36,37]. In the BALF, a marked increase in TNF-α and in IL-8 was noted after LPS inhalation when compared to cytokine concentrations determined as being close to the detection limit in group of unexposed healthy volunteers [23]. After LPS inhalation, TNF-α concentration was elevated at 8 h and fell after 24 h in all infusion groups. The IL-8 concentrations 8 and 24 h after LPS inhalation were considerably higher than in unexposed volunteers in the present study, and similar data have been reported by others [23,37]. When comparing the effect of infusion therapy on cytokine generation a threefold increase in TNF-α concentration in the SO group compared to the NaCl group after 8 h and a 50 % reduction in IL-8 in the FO group compared to this group after 24 h were visible. Most data available on effects of n-3 and n-6 lipids point to a reduction in inflammatory reactions by FO-derived lipids [9,18,32]. The finding of a pro-inflammatory effect of n-6 lipids is more difficult to document [18,33].

### Onset and persistence of the effect of lipid emulsions

The differential effect of lipid emulsions on isolated monocytes or neutrophil cytokine generation required pre-infusion and was measurable after only a two-day infusion course and sustained up to 24 h after cessation of the infusion, whereas the effect on adhesion and rolling of monocytes was found at 8 h, but not at 24 h. This finding is confirmed by the modulation of TNF-α and IL-8 concentrations in the BALF by the lipid emulsions after 8 h or 24 h, respectively. In contrast to a previous study in volunteers [18] where the effect of lipid emulsions was observed in isolated monocytes 2 h after termination of the infusion period, persistence of the effect of a short-term 48-h infusion period for 24 h after termination of the infusion was confirmed in the present study.

### Impact of lipid emulsions on the inflammatory response

Infusion of conventional (SO) or FO-based lipid emulsions may not only regarded as a source of calories for patients with a need for parenteral nutrition as these lipid emulsions also modify the inflammatory response. This is the first study in human volunteers to show that after subsequent standardized LPS inhalation, the ability of lipid emulsions to change adhesive properties of leukocytes, including the generation of cytokines translating into a modified invasion of leukocytes into the alveolar space. After infusion of FO-based emulsions, recruited neutrophils in the alveolar space were reduced. In contrast, rolling and adhesion of monocytes, and TNF-α concentration in BALF were increased in the group receiving conventional (SO-based) lipid emulsions. Both lipid emulsions differ only in the fatty acids supplied and are used in many countries worldwide; however, both may not be regarded as immune-neutral. It may be of interest for the choice of lipid emulsions when considering parenteral infusions. Currently, the use of lipid emulsions rich in n-6 lipids is not encouraged by European Guidelines [38]. This is in line with a recent Spanish multicenter study demonstrating a higher infection rate in critically ill patients receiving a standard lipid emulsion as compared to a lipid emulsion enriched with FO [15]. For our study, we chose a pre-treatment strategy by administration of lipid emulsions prior to induction of pulmonary inflammation. This concept is of clinical relevance as we might pre-treat/pre-condition patients with an expected trauma (for example, operation) with lipid emulsion to modulate an immune response. Thus far there is a paucity of data investigating this concept in terms of FO-based lipid emulsions and inflammation/sepsis. Augmenting the line of our reasoning, a randomized controlled trial in surgical patients found that pre-operative intravenous application of an FO-based lipid emulsion modulated the post-operative immune response [39].

The involvement of resolvins as a novel class of n-3 fatty acid derived lipid mediators might be responsible for the different effects observed after infusion of FO or SO in the context of ALI. This is the first study elucidating the impact of the resolvin E1 receptor ChemR23 on the pathogenesis of ARDS and lipid mediators. ChemR23−/− mice show a diverse behavior compared to WT controls, especially when challenged with FO, this novel pathophysiologic hint appears promising and deserves further investigation. Furthermore, the difference between WT and ChemR23−/− mice is quite striking and reinforces a role for the ChemR23-endogenous chemerin pathway in the modulation of the innate immune response in vivo.

## Conclusion

The present study extends previous observations in demonstrating that a 48-h lipid infusion period suffices to modify leukocyte adhesive properties as well as cytokine generation and leukocyte invasion into the alveolar space. This observation is of major interest as both lipid emulsion employed in this investigation are approved for parenteral nutrition in many countries. When considering parenteral nutrition in stable patients, for example, before an operation, use of lipid emulsions enriched in n-6 lipids does not seem to be beneficial. In fact, meta-analysis of surgical patients and intensive care patients undergoing parenteral nutrition supports the view that including a small fraction of n-3 lipids into parenteral regimes did reduce secondary infections and shortened the length of stay [40,41]. The findings of this study leave no doubt that in addition to fulfilling nutritional functions, intravenous administration of lipid emulsions has a strong impact on immunological functions and inflammatory processes in humans.

## Key messages

- Lipid emulsion commercially available for clinical use exerts different immunological effects on alveaolar recruitment of leukocytes and generation of cytokines in human volunteers
- FO-based lipid emulsions appear to reduce alveolar recruitment of leukocytes and levels of pro-inflammatory cytokines after LPS challenge.
- After infusion of SO-based lipid emulsions a predominantly pro-inflammatory pattern is observed
- Beneficial effects of FO might at least in part be mediated by resolvinE1 via ChemR23 receptor

## Additional file

Additional file 1:
**Immunomodulation and lipid emulsions.**

## Abbreviations

ALI: acute lung injury
ANOVA: analysis of variance
ARDS: acute respiratory distress syndrome
BAL: bronchoalveolar lavage
BALF: bronchoalveolar lavage fluid
CCR: CC-chemokine receptor
ChemR: chemerin receptor
EDTA: ethylenediaminetetraacetic acid
ELISA: enzyme-linked immunosorbent assay
FO: fish oil
IL: interleukin
LPS: lipopolysaccharide
MIP: macrophage inflammatory protein
PMN: polymorphonuclear granulocytes
PSGL: P-selectin glycosylated ligand
RT-PCR: reverse-transcription polymerase chain reaction
RvE1: Resolvin E1
SO: soybean oil
TNF-α: tumor-necrosis factor-α
Tx: thromboxane
