# Supplementary material for: Immunomodulation by lipid emulsions in pulmonary inflammation: a randomized controlled trial
Source: Crit Care. 2015 May 12;19(1):226. doi: 10.1186/s13054-015-0933-6 (PMC4438480; doi:10.1186/s13054-015-0933-6)
Supplement: Additional file 1: — Immunomodulation and lipid emulsions. [file 13054_2015_933_MOESM1_ESM.pdf]

Supplemental material:

Immunomodulation by lipid emulsions in pulmonary inflammation: a randomized controlled trial

Matthias Hecker[1], Tomke Linder[1], Juliane Ott[1], Hans-Dieter Walmrath[1], Jürgen Lohmeyer[1], Istvan Vadasz[1], Leigh M. Marsh[1], Susanne Herold[1], Martin Reichert[1], Anja Buchbinder[1], Rory E. Morty[2], Britta Bausch[1], Tobias Fischer[1], Richard Schulz[1], Friedrich Grimminger[1], Martin Witzenrath[3], Matt Barnes[4], Werner Seeger[1], and Konstantin Mayer[1]

[1]University of Giessen and Marburg Lung Center (UGMLC), Justus-Liebig-University of Giessen, Klinikstr. 33, D – 35392 Giessen, Germany; [2] Department of Lung Development and Remodelling, Max Planck Institute for Heart and Lung Research, Bad Nauheim, Germany; [3]Charité - Universitätsmedizin Berlin, Medizinische Klinik mit Schwerpunkt Infektiologie und Pneumologie, Berlin, Germany, [4] Takeda Cambridge Ltd., Cambridge, UK.

Address correspondence to:
Dr. Konstantin Mayer
University of Giessen and Marburg Lung Center (UGMLC)
Medical Clinic II, Klinikstr. 33, 35392 Giessen, Germany
Tel.: +49-641-985-56743, Fax.: +49-641-985-51259
E-mail: Konstantin.Mayer@innere.med.uni-giessen.de

Supported by: Bundesministerium für Bildung und Forschung, German Center for Lung Research (DZL); Transregio 84 “Innate Immunity of the Lung: Mechanisms of Pathogen Attack and Host Defence in Pneumonia”, Project C3.

Keywords: fish oil, inflammation, ARDS, lipid emulsions, resolvin, ChemR23

Material and Methods:

Volunteer selection.

The volunteers were >18 years of age, did not smoke, and were not vegetarians. They did not take fish oil capsules or any comparable nutritional supplementation. They were evaluated as to be completely healthy after a complete history, an ECG, normal routine laboratory including differential blood count, coagulation parameters, electrolytes, liver and pancreas enzymes, renal parameters, creatinine kinase, lipid status, urine test and physical examination performed by physicians not related to our facilities. Volunteers with a history or current episode of metabolic disorder (especially diabetes or lipid disorders), gastro-intestinal, pulmonary, cardio-vascular, hematological, allergic, rheumatological, or immunological disease were not accepted. The volunteers did not take medication for at least six weeks before the start of the infusions. Volunteers were screened to have a normal pulmonary function test using a standard body-plethysmograph and did not exhibit a bronchial hyperreactivity after metacholine challenge. Of 115 volunteers screened, 42 were included in the study. Exclusion criteria were abnormal routine laboratory values (n = 27), abnormal pulmonary function test (n = 35), and abnormal response to inhalative metacholine provocation (n = 11).

Data from four volunteers were not available for evaluation as one volunteer withdrew during the infusion course, two volunteers withdrew before bronchoscopy, and a third volunteer was detected to be a smoker before undergoing bronchoscopy. Thirty-eight volunteers (23 male) were entered into data-analysis. The mean age was 29.7 years (range 19 – 56) and a mean body mass index of 23.4 (range 18.1 – 31.0).

LPS inhalation and bronchoalveolar lavage

The LPS inhalation was carried was carried out as described by Kline and coworkers (1) using an AKITA® inhalation device from inamed (Gemünden, Germany) which allows

smartcard-aided control of compliance and deposition. All volunteers were exposed by inhalation challenge to buffered sterile saline (HBSS) followed by increasing concentrations of LPS (*E. coli* (0111:B4; Sigma, Munich, Germany)). After the HBSS, subsequent inhalations delivered step-wise increasing doses of LPS according to the following schedule: 0.5 mg, 1.0 mg, 2.0 mg, 3.0 mg, 5.0 mg, 10 mg, and 20 mg. Inhalation of all doses would result in a of 41.5 mg of LPS. A spirometer was used to assess pulmonary function before and after LPS-challenge. Volunteers were sitting upright in a chair and were using noseclips. Baseline spirometry was recorded before inhalation of saline, and then 1, 10, 20, and 30 min after each inhalation of HBSS or each dose of LPS, and compared with the pre-saline baseline. If a volunteer's forced expiratory volume in the first second (FEV1) was >80% of the baseline determination, the inhalation challenge was continued and the next dose of LPS was inhaled. The LPS-challenge was terminated when either (*i)* the subject did not wish to continue for any reason; (*ii*) the volunteer's FEV1 decreased 20% or greater from baseline; or (*iii*) a cumulative dose of 41.5 mg LPS had been achieved. The cumulative dose of LPS to result in a reduction of 20% or greater in FEV1 was considered as the specific dose of each volunteer. Cumulative doses were 8.1 ± 1.5 µg, 14.6 ± 3.9 µg, and 8.7 ± 1.4 µg in the NaCl, FO, and SO group respectively ($p = 0.382$, not significant). Twelve weeks after the first LPS-inhalation and after completion of the infusion course, the volunteers were re-exposed to their individual dose in two inhalation courses. They were randomized to undergo bronchoscopy and bronchoalveolar lavage of either segment 4 or 5 of the right lung 8 or 24 h after completion of the inhalative LPS-challenge. Lavage was carried our using 150 ml of pre-warmed NaCl 0.9 %.

Supplement, Fig. E1:

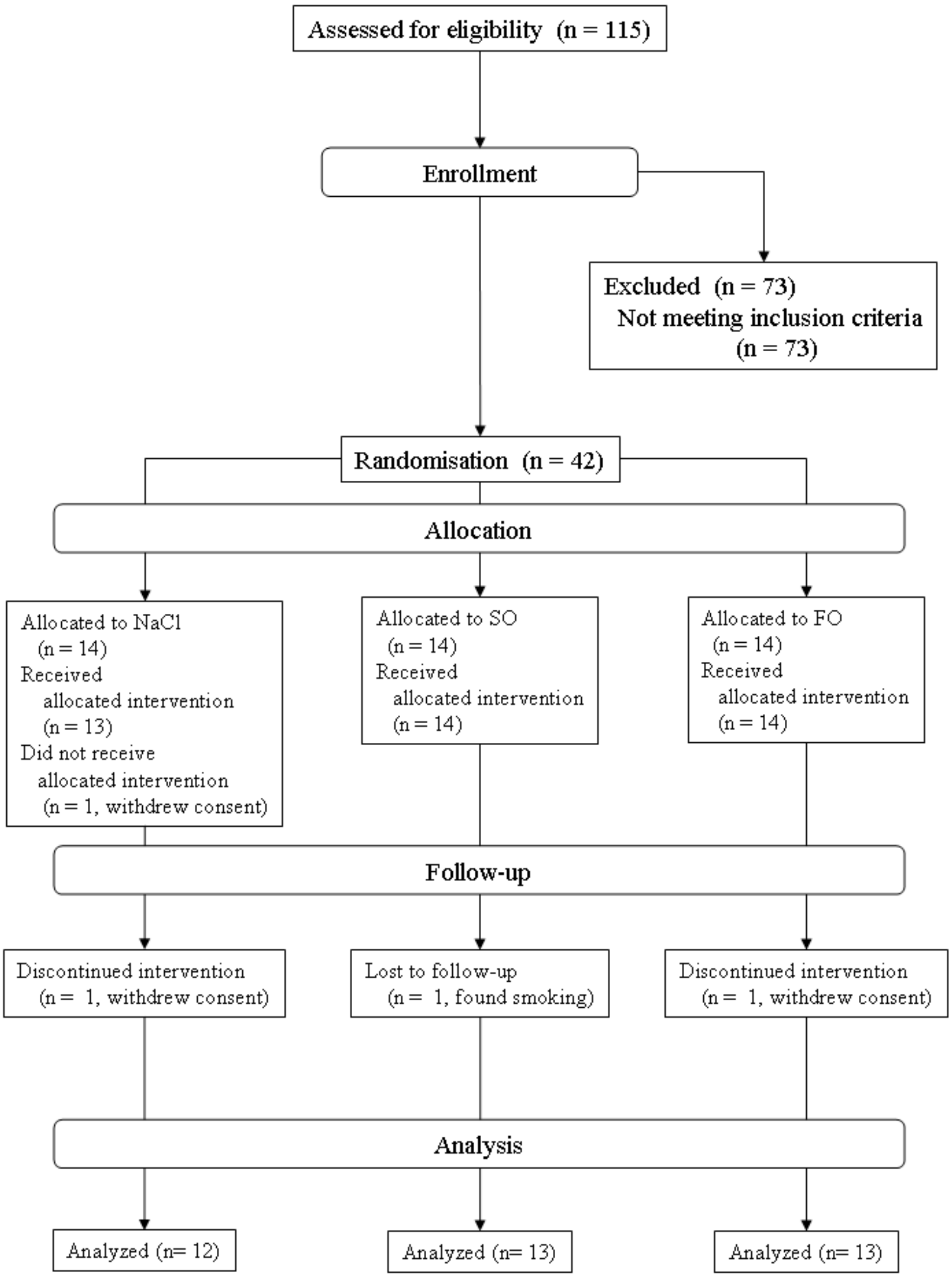

Fig E1 : Flow of participants through the study.

Volunteers were screened and randomized to one of the three infusion treatments (normal saline, NaCl; fish oil-based lipid emulsion, FO; soybean oil-based lipid emulsion, SO).

Supplement, Fig. E2)

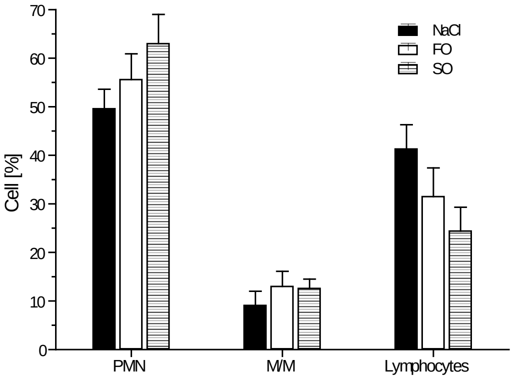

a) Differentiation at 8h

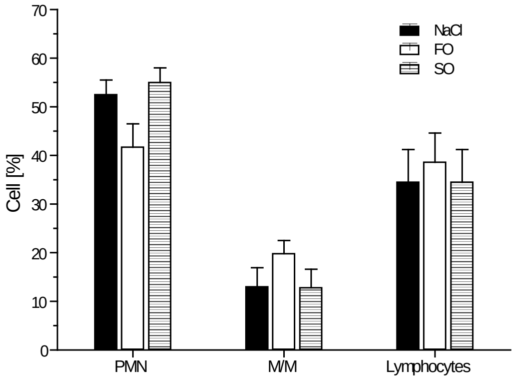

b) Differentiation at 24h

Supplement Fig. E2: Leukocyte differentiation in the bronchioalveolar lavage fluid

Volunteers received either normal saline (NaCl), a fish oil (FO)-, or a soybean oil (SO)-based lipid emulsion before bronchioalveolar lavage was performed 8 (a) or 24 h (b) after inhalation of LPS. Leukocytes were differentiated into neutrophils (PMN), monocytes and macrophages (M/M), or lymphocytes. Numbers are given as mean ± SEM, n = 6 – 7 volunteers per time point and group.

Supplement, Fig. E3)

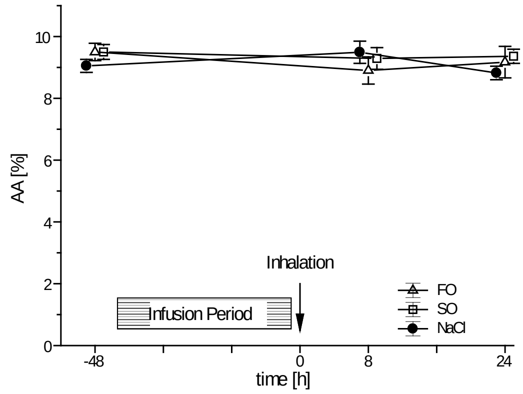

a) Arachidonic acid in PMN

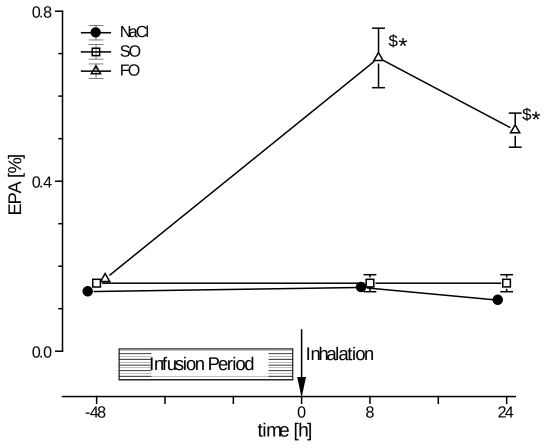

b) Eicosapentaenoic acid in PMN

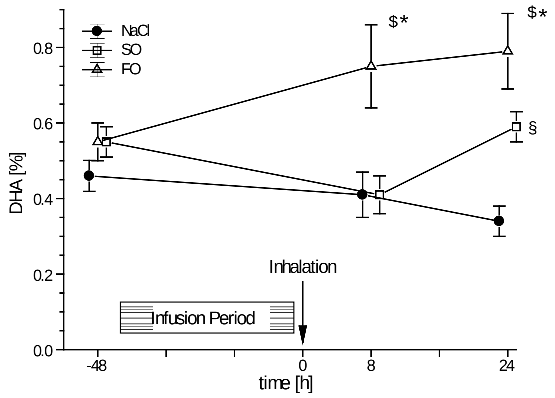

c) Docosahexaenoic acid in PMN

Supplement Fig. E3: Fatty acids in the PMN membranes

Isolated PMN originated from volunteers receiving fish oil (FO)- or soybean oil (SO)-based lipid infusion, or normal saline (NaCl). Membrane fatty acids (a – c) were determined

by gas chromatography. Arachidonic acid (AA, a) levels remained constant in all groups. Eicosapentaenoic acid (EPA, b) and docosahexaenoic acid (DHA, c) levels increased in the FO group (*, $p < 0.05$ vs. baseline, \$, $p < 0.05$ vs. SO and NaCl). At 24 h post inhalation, DHA in the SO group differed from the NaCl group (§, $p < 0.05$).

Tables:

Table E1: Fatty acid composition of the n-6 and the n-3 lipid-emulsion (g/l).

Fatty acid (g/l)	n-6 lipid emulsion	n-3 lipid emulsion
C14:0	-	4.9
C16:0	12.4	10.7
C16:1 n-7	-	8.2
C18:0	5.0	2.4
C18:1 n-9	24.1	12.3
C18:2 n-6	52.2	3.7
C18:3 n-3	8.2	1.3
C20:4 n-6	-	2.6
C20:5 n-3	-	18.8
C22:5 n-3	-	2.8
C22:6 n-3	-	16.5
Others	-	16.1

The n-6 (Lipoven®) and the n-3 lipid-emulsion (Omegaven®) were manufactured with identical techniques and additives. Repetitive gas chromatographic controls of both lipid emulsions revealed less than 0.3% free EPA or AA as related to the esterified amounts of these fatty acids.

Table E2: Surface Adhesion Molecules in isolated Monocytes

**Surface Molecule**	**Group** / **Time**	**Baseline**	**8 h post LPS-inhalation**	**24 h post LPS-inhalation**
CD11b	**NaCl**	**435.7 ± 8.1 (n = 12)**	**460.3 ± 14.2 (n = 7)** [aa]	409.5 ± 5.1 (n = 5) [bb]
	**FO**	**442.1 ± 12.1 (n = 12)**	**507.8 ± 10.8 (n = 7)** [aa ,11]	448.4 ± 19.1 (n = 6) [bb, 2]
	**SO**	**419.8 ± 15.8 (n = 11)**	**472.2 ± 13.6 (n = 7)** [aa, 1]	443.0 ± 23.4 (n = 6) [bb]
CD18	**NaCl**	**292.1 ± 9.4 (n = 12)**	**307.2 ± 13.7 (n = 7)** [aa]	287.5 ± 9.6 (n = 5)
	**FO**	**283.2 ± 6.7 (n = 12)**	**330.7 ± 14.9 (n = 7)** [aa, 1]	315.0 ± 17.9 (n = 6)
	**SO**	**288.5 ± 13.8 (n = 11)**	**328. 2 ± 15.9 (n = 7)** [aa]	295.9 ± 14.1 (n = 6)
CD49d	**NaCl**	**295.8 ± 11.8 (n = 12)**	**292.1 ± 7.9 (n = 6)** [a]	333.2 ± 11.9 (n = 4) [bb]
	**FO**	**304.3 ± 9.0 (n = 12)**	**263.6 ± 17.0 (n = 7)** [a]	295.6 ± 9.3 (n = 6) [bb]
	**SO**	**297.5 ± 12.6 (n = 12)**	**272.9 ± 13.0 (n = 7)** [a]	328.0 ± 22.1 (n = 6) [bb, 2]

Surface Molecule / Group \ Time		Baseline	8 h post LPS-inhalation	24 h post LPS-inhalation
CD62L	**NaCl**	**261.2 ± 13.2 (n = 12)**	**270.9 ± 18.6 (n = 7)**	297.7 ± 23.7 (n = 5)
	**FO**	**252.0 ± 10.5 (n = 13)**	**299.0 ± 18.4 (n = 7)**	232.6 ± 26.6 (n = 6)
	**SO**	**251.8 ± 10.8 (n = 13)**	**266.4 ± 16.3 (n = 7)**	281.9 ± 15.7 (n = 6)
CD162	**NaCl**	**438.6 ± 11.7 (n = 10)**	**420.4 ± 12.9 (n = 5)**	456.5 ± 9.2 (n = 5)
	**FO**	**420.2 ± 13.8 (n = 12)**	**446.4 ± 9.1 (n = 7)**	401.2 ± 26.2 (n = 6)
	**SO**	**414.4 ± 17.8 (n = 11)**	**427.9 ± 16.1 (n = 6)**	422.9 ± 26.6 (n = 6)
CD14	**NaCl**	**609.4 ± 6.7 (n = 12)**	**615.8 ± 7.9 (n = 7)**	611.9 ± 7.8 (n = 5)
	**FO**	**611.1 ± 5.6 (n = 12)**	**624.5 ± 7.4 (n = 7)**	612.8 ± 15.3 (n = 6)
	**SO**	**607.8 ± 8.1 (n = 13)**	**630.3 ± 5.7 (n = 7)**	631.5 ± 10.6 (n = 6)
CD45	**NaCl**	**476.3 ± 5.9 (n = 12)**	**477.6 ± 8.4 (n = 7)**	486.4 ± 9.2 (n = 5)

Surface Molecule / Group / Time		Baseline	8 h post LPS-inhalation	24 h post LPS-inhalation
	**FO**	**477.5 ± 5.7 (n = 13)**	**488.3 ± 4.6 (n = 7)**	461.5 ± 18.5 (n = 6)
	**SO**	**473.1± 4.1 (n = 13)**	**492.3 ± 5.8 (n = 7)**	489.7 ± 6.9 (n = 6)
CCR2	**NaCl**	**306.6 ± 10.1 (n = 12)**	**295.1 ± 16.1 (n = 7)**	314.0 ± 21.0 (n = 5)
	**FO**	**312.6± 10.5 (n = 13)**	**306.4 ± 8.5 (n = 7)**	320.9 ± 10.5 (n = 6)
	**SO**	**311.0 ± 3.3 (n = 13)**	**307.9 ± 11.8 (n = 7)**	321.4 ± 12.5 (n = 6)
CCR5	**NaCl**	**37.0 ± 12.5 (n = 12)**	**29.5 ± 12.6 (n = 7)**	72.1 ± 15.9 (n = 5)
	**FO**	**30.4 ± 8.3 (n = 13)**	**58.7 ± 11.0 (n = 7)**	35.6 ± 14.2 (n = 6)
	SO	27.4 ± 8.5 (n = 13)	29.2 ± 8.6 (n = 7)	46.9 ± 17.8 (n = 6)

Effect of time: a, $p < 0.05$ vs. baseline; aa, $p < 0.01$ vs. baseline; b, $p < 0.05$ vs. 8 h; bb, $p < 0.01$ vs. 8 h.

Effect of time within infusion groups: 1, $p < 0.05$ vs. baseline; 11, $p < 0.01$ vs. baseline; 2, $p < 0.05$ vs. 8 h; 22, $p < 0.01$ vs. 8 h.

Table E3: Surface Adhesion Molecules in isolated PMN

Surface Molecule	Group \ Time	Baseline	8 h post LPS-inhalation	24 h post LPS-inhalation
CD11b	**NaCl**	**484.7 ± 12.7 (n = 12)**	**495.4 ± 26.3 (n = 7)**	433.7 ± 24.8 (n = 5) [a, b]
	**FO**	**535.7 ± 15.6 (n = 13)**	**564.1 ± 18.5 (n = 7)**	486.6 ± 27.7 (n = 6) [a, b, 2]
	**SO**	**515.7 ± 18.0 (n = 12)**	**499.4 ± 27.6 (n = 7)**	498.8 ± 16.6 (n = 6) [a, b]
CD18	**NaCl**	**338.0 ± 7.8 (n = 10)**	**332.1 ± 19.4 (n = 7)**	303.0 ± 17.6 (n = 6)
	**FO**	**355.6 ± 7.8 (n = 13)**	**369.8 ± 19.0 (n = 7)**	337.8 ± 15.9 (n = 6)
	**SO**	**347.9 ± 16.5 (n = 12)**	**353.0 ± 16.9 (n = 5)**	327.7 ± 14.0 (n = 6)
CD49d	**NaCl**	**55.3 ± 7.2 (n = 11)**	**60.6 ± 11.9 (n = 7)**	50.8 ± 11.6 (n = 4)
	**FO**	**57.6± 7.4 (n = 13)**	**66.5 ± 11.5 (n = 6)**	63.3 ± 14.1 (n = 4)

**Surface Molecule**	**Time** / **Group**	**Baseline**	**8 h post LPS-inhalation**	**24 h post LPS-inhalation**
	**SO**	**55.8 ± 6.0 (n = 13)**	**57.4 ± 8.5 (n = 7)**	63.9 ± 6.8 (n = 5)
CD62L	**NaCl**	**311.4 ± 10.8 (n = 12)**	**306.9 ± 16.2 (n = 7)**	302.3 ± 19.6 (n = 5)
	**FO**	**307.0 ± 15.7 (n = 13)**	**319.2 ± 12.5 (n = 7)**	287.1 ± 20.2 (n = 6)
	**SO**	**313.8 ± 11.7 (n = 13)**	**307.5 ± 9.1 (n = 7)**	298.2 ± 10.6 (n = 6)
CD162	**NaCl**	**465.3 ± 9.9 (n = 11)**	**443.8 ± 17.1 (n = 5)**	453.1 ± 9.5 (n = 5)
	**FO**	**459.6 ± 8.7 (n = 11)**	**464.5 ± 9.4 (n = 7)**	418.5 ± 18.1 (n = 6)
	**SO**	**458.2 ± 15.6 (n = 10)**	**413.9 ± 29.2 (n = 7)**	424.8 ± 20.0 (n = 5)
CD14	**NaCl**	**114.0 ± 9.5 (n = 11)**	**159.9 ± 14.8 (n = 6)** [aa]	162.4 ± 9.2 (n = 5) [aa]
	**FO**	**135.7± 8.6 (n = 11)**	**192.0 ± 14.3 (n = 7)** [aa, 11]	235.2 ± 23.6 (n = 5) [aa, 11, **]

Surface Molecule	Group / Time	Baseline	8 h post LPS-inhalation	24 h post LPS-inhalation
	**SO**	**144.8 ± 8.2 (n = 11)**	**212.6 ± 35.7 (n = 4)** [aa, 11]	242.3 ± 23.7 (n = 6) [aa, 11, **]
CD45	**NaCl**	**380.0 ± 3.7 (n = 11)**	**366.8 ± 14.3 (n = 7)**	376.5 ± 10.9 (n = 5)
	**FO**	**405.1 ± 9.2 (n = 13)**	**402.9 ± 10.6 (n = 7)**	375.9 ± 22.4 (n = 6)
	**SO**	**394.6 ± 8.6 (n = 13)**	**382.6 ± 14.4 (n = 7)**	400.6 ± 10.5 (n = 6)
CCR2	**NaCl**	**11.2 ± 3.1 (n = 12)**	**9.1 ± 4.6 (n = 7)**	15.3 ± 5.6 (n = 5)
	**FO**	**15.9 ± 3.6 (n = 13)**	**11.6 ± 4.5 (n = 7)**	24.0 ± 10.9 (n = 6)
	**SO**	**15.3 ± 2.3 (n = 13)**	**14.3 ± 3.8 (n = 7)**	12.8 ± 4.9 (n = 6)
CCR5	**NaCl**	**45.8 ± 3.9 (n = 12)**	**40.8 ± 3.4 (n = 7)**	63.2 ± 8.0 (n = 5)
	**FO**	**54.6 ± 5.9 (n = 13)**	**41.0 ± 10.5 (n = 7)**	54.1 ± 7.4 (n = 6)

Surface Molecule	Group (Time)	Baseline	8 h post LPS-inhalation	24 h post LPS-inhalation
	SO	27.4 ± 8.5 (n = 13)	29.2 ± 8.6 (n = 7)	46.9 ± 17.8 (n = 6)

Effect of time: a, $p < 0.05$ vs. baseline; aa, $p < 0.01$ vs. baseline; b, $p < 0.05$ vs. 8 h; bb, $p < 0.01$ vs. 8 h.

Effect of time within infusion groups: 1, $p < 0.05$ vs. baseline; 11, $p < 0.01$ vs. baseline; 2, $p < 0.05$ vs. 8 h; 22, $p < 0.01$ vs. 8 h.

Effect of infusion groups within time: *, $p < 0.05$ vs. NaCl; **, $p < 0.01$ vs. NaCl.

References

1. Kline JN, Cowden JD, Hunninghake GW, et al. Variable airway responsiveness to inhaled lipopolysaccharide. Am J Respir Crit Care Med 1999;160(1):297-303.
